# Coxsackievirus B4 Transplacental Infection Severely Disturbs Central Tolerogenic Mechanisms in the Fetal Thymus

**DOI:** 10.3390/microorganisms9071537

**Published:** 2021-07-19

**Authors:** Aymen Halouani, Hélène Michaux, Habib Jmii, Charlotte Trussart, Ahlem Chahbi, Henri Martens, Chantal Renard, Mahjoub Aouni, Didier Hober, Vincent Geenen, Hela Jaïdane

**Affiliations:** 1 Laboratoire des Maladies Transmissibles et Substances Biologiquement Actives LR99ES27, Faculté de Pharmacie de Monastir, Université de Monastir, Monastir 5000, Tunisia; halouani.aymen@yahoo.com (A.H.); jmiihbib@yahoo.fr (H.J.); aouni_mahjoub2005@yahoo.fr (M.A.); 2 Faculté des Sciences de Tunis, Université de Tunis El Manar, Tunis 1068, Tunisia; 3 GIGA-I3 Immunoendocrinologie, Faculté de Médicine, Université de Liège, CHU-B34, Sart Tilman, 4000 Liège, Belgium; hmichaux88@gmail.com (H.M.); charlotte.trussart@uliege.be (C.T.); hmartens@uliege.be (H.M.); ch.renard@chu.ulg.ac.be (C.R.); vgeenen@uliege.be (V.G.); 4 Laboratoire d’Hématologie, Faculté de Médecine de Tunis, Université de Tunis El Manar, Tunis 1007, Tunisia; chahbi.a7lem@gmail.com; 5 Laboratoire de Virologie EA3610, Faculté de Médecine, Université de Lille, CHU Lille, 59000 Lille, France; didier.hober@univ-lille.fr

**Keywords:** Coxsackievirus B4, in utero infection, thymus, thymic epithelial cells, central self-tolerance, transcription factors, autoantigens, autoimmunity

## Abstract

Thymus plays a fundamental role in central tolerance establishment, especially during fetal life, through the generation of self-tolerant T cells. This process consists in T cells education by presenting them tissue-restricted autoantigens promiscuously expressed by thymic epithelial cells (TECs), thus preventing autoimmunity. Thymus infection by Coxsackievirus B (CV-B) during fetal life is supposed to disturb thymic functions and, hence, to be an inducing or accelerating factor in the genesis of autoimmunity. To further investigate this hypothesis, in our current study, we analyzed thymic expression of autoantigens, at the transcriptional and protein level, following in utero infection by CV-B4. mRNA expression levels of *Igf2* and *Myo7*, major autoantigens of pancreas and heart, respectively, were analyzed in whole thymus and in enriched TECs together along with both transcription factors, *Aire* and *Fezf2*, involved in autoantigens expression in the thymus. Results show that in utero infection by CV-B4 induces a significant decrease in *Igf2* and *Myo7* expression at both mRNA and protein level in whole thymus and in enriched TECs as well. Moreover, a correlation between viral load and autoantigens expression can be observed in the whole thymus, indicating a direct effect of in utero infection by CV-B4 on autoantigens expression. Together, these results indicate that an in utero infection of the thymus by CV-B4 may interfere with self-tolerance establishment in TECs by decreasing autoantigen expression at both mRNA and protein level and thereby increase the risk of autoimmunity onset.

## 1. Introduction

The thymus is an organ located in the anterior mediastinum just above the heart. It is flattened, bilobed, and its principle role is the generation of mature T lymphocytes. It can be divided into cortical and medullar regions, which are delimited by the corticomedullary junction [1,2]. T lymphocytes, prior to being released into the circulation, must undergo both positive and negative selection [3,4]. The first selection occurs in the cortex and consists in the elimination of T cell precursors having a T cell receptor (TCR) with low affinity to the Major Histocompatibility Complex (MHC) molecule (and preservation of those with good affinity) [5]. During negative selection, autoantigens, representing each body tissue and promiscuously expressed by medullar thymic epithelial cells (mTEC), will be presented by antigen presenting cells (APC) in a view to educate T cells to tolerate their own body components, through the elimination of self-reactive T cells [6]. The expression of those tissue-restricted autoantigens (TRAs) is essentially regulated by two transcription factors, namely *Aire* (*autoimmune regulator*) and *Fezf2* (*forebrain expressed zinc finger 2*), playing a critical role in thymic negative selection [7].

Coxsackievirus B (CV-B) are ubiquitous and widespread viruses, belonging to the species Enterovirus B, of the genus Enterovirus, of the family *Picornaviridae*. CV-Bs infections are considered to be mild, nevertheless, they are also frequently implicated in various acute and chronic pathologies, some of which have an autoimmune component, such as myocarditis (reviewed by [8]), type 1 diabetes (T1D) (reviewed by [9]), Sjögren’s syndrome [10], and certain neurological diseases (reviewed by [11]).

Autoimmunity is a process in which the body does not recognize its own components, which leads to their destruction and attack by the immune system. This phenomenon is the result of complex interactions between genetic, immunological, hormonal, and environmental factors. Although autoimmune diseases usually have an asymptomatic beginning, environmental factors, especially viral infection, can initiate an overt expression [12,13]. Their common denominator is the loss of tolerance to autoantigens, especially to those widely expressed in the thymus during fetal life and a few days after birth, which manifests through the appearance of autoreactive T cells that have escaped negative selection [13].

Numerous studies show that genetic or acquired dysfunction of the thymus in the programming of central immune tolerance towards the Self, plays a decisive role in the development of organ-specific autoimmune diseases (reviewed by [14]). More known as a privileged immunological site, protected from infection and immune response, it is now clearly recognized that the thymus is targeted by various pathogens, particularly viruses (reviewed by [15,16]), able to alter both the structure and function of this primary lymphoid organ (reviewed by [16,17]). These alterations are intimately related to one another and will generally impact the consequences of the infection in the long term.

Among the six CV-B serotypes, CV-B4 seems to be the most involved in the pathogenesis of T1D (reviewed by [9]). Thus, this virus is the most used in experimental studies trying to elucidate the physiopathological mechanisms leading to T1D (reviewed by [18]).CV-B4 E2, most known as the diabetogenic strain, but revealed with a wide tropism [19,20], can infect mouse total thymic cells in vitro [21], human and murine thymic epithelial cells [22,23], as well as human and murine fetal thymus organ cultures [24,25]. A persistent decrease in the expression of insulin-like growth factor 2 *(Igf2*), a major autoantigen of the pancreas, in a cell line of murine thymic epithelial cells infected with CV-B4 E2, has already been documented [23]. In utero infection of the mouse thymus by CV-B4 E2 was recently documented, and significant anomalies of the different thymocyte populations were revealed [26]. Altogether, these data considerably reinforce the hypothesis of the role of CV-B4 infection of the thymus, especially in the fetal and neonatal life, in the development of autoimmune pathologies.

The critical issue of possible fetal thymus involvement through vertical transmission of CV-B and on the molecular mechanisms involved in the pathogenesis of autoimmune diseases has been very poorly documented [27,28].

In our current study, we aim to evaluate the expression of autoantigens representing target tissues during CV-B infections, namely *Igf2* for the pancreas and myosin 7 (*Myo7*) for the heart, together with both *Aire* and *Fezf2* transcription factors in the whole thymus and enriched TECs, which we also considered in our recently described murine model of in utero CV-B4 infection.

## 2. Materials and Methods

### 2.1. Virus

The CV-B4 E2 strain (kindly provided by J. W. Yoon, Julia McFarlane Diabetes Research Centre, Calgary, AB, Canada) was propagated in human epithelial type 2 (HEp-2) cells (BioWhittaker, Walkersville, MD, USA) in Eagle’s minimum essential medium (MEM; GIBCO BRL, Invitrogen, Gaithersburg, MD, USA) supplemented with 10% heat-inactivated fetal calf serum (FCS; GIBCO BRL), 1% (2 mM) l-glutamine (BioWhittaker), 1% non-essential amino-acids (GIBCO BRL), 50 µg/mL streptomycin, 50 IU/mL penicillin (GIBCO BRL), and 0.05% (2.5 µg/mL) fungizone (Amphotericin B; Apothecon, Amsterdam, The Netherlands). Supernatants were collected 3 days post-inoculation (p.i.), clarified by centrifugation at 4000× *g* for 10 min, divided into aliquots and stored at −80 °C. Virus titers were determined on HEp-2 cells by limiting dilution assay for 50% tissue culture infectious doses (TCID_50_) by the method of Reed and Muench [29].

### 2.2. Mice

Adult outbred *Swiss albino* mice (Pasteur Institute, Tunis, Tunisia) handled in the animal facility of the Faculty of Pharmacy of Monastir, were used in this investigation. All experiments were performed by following the standards of general ethics guidelines and approved by the bioethics committee in the Higher Institute of Biotechnology of Monastir, University of Monastir, Tunisia. Mice were housed with ad libitum access to food pellets and tap water, and kept under controlled conditions. Mice were mated (four females per male were caged together) until successful fertilization was noted. The day the vaginal plug was observed was considered as the first day of gestation (day 1G).

### 2.3. Mice Inoculation and Follow-Up

**Figure 1:**
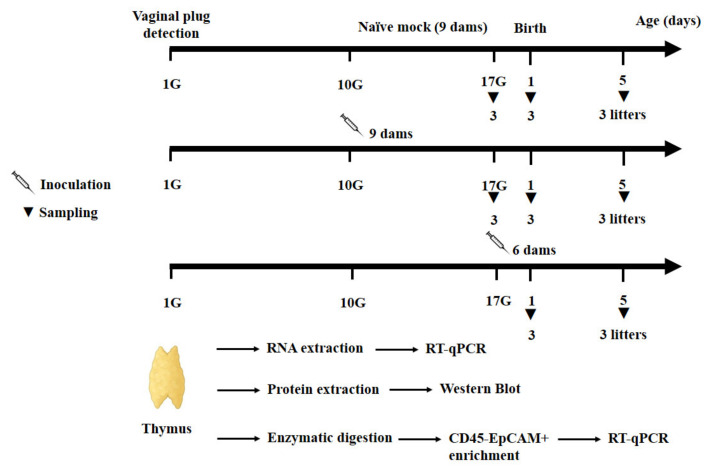
Schematic representation of the experimental schedule adopted to explore the effect of in utero CV-B4 infection on transcription factors and autoantigens expression. Four mice females per male were caged together until successful fertilization. The day of detection of the vaginal plug is considered as the first day of gestation (day 1G). Pregnant mice were orally inoculated with CV-B4 E2, randomly, either at gestational days 10 (10G, 9 dams) or 17 (17G, 6 dams). Nine naive pregnant dams served as mock-infected negative controls. Offspring’s thymuses were harvested at day 17G, day 1, and day 5 from birth when mice were Mock- or CV-B4-inoculated at day 10G, and at day 1 and 5 from birth when mice were CV-B4-inoculated at day 17G. For each experimental condition (Mock- or CV-B4-inoculated at day 10G or 17G) and each sampling point (day 17G and days 1 and 5 from birth), thymuses were sampled from three litters of mice.

Pregnant mice were inoculated randomly, either at gestational day 10 (day 10G, 9 dams) or 17 (day 17G, 6 dams), orally (by gavage) with 2 × 10^6^ TCID_50_ of CV-B4 E2 contained in 200 µL culture medium. Age-matched offspring (fetuses and new-born pups) from nine naive pregnant mice served as mock-infected negative controls. At each of the different time-points (day 17G, and days 1 and 5 from birth), three litters of mice from each experimental condition (Mock- or CV-B4-inoculated at day 10G or 17G) were killed using isoflurane (Zoetis) and offspring’s thymuses were sampled (Figure 1). All samples were washed with cold phosphate-buffered saline (PBS) and stored at −80 °C until RNA or protein extraction.

### 2.4. TECs Isolation and Immunostaining

Thymic lobes were harvested from mice of the same litter for each group, washed in cold PBS and transferred into cold DMEM (Invitrogen, Waltham, MA, USA) supplemented with 10% heat-inactivated FCS (Invitrogen, Waltham, MA, USA), 20 mM HEPES, 50 µg/mL streptomycin, 50 U/mL penicillin, and 0.05% (2.5 µg/mL) fungizone. Thymuses were cut into small pieces and fragments were dispersed further via pipetting to remove the majority of thymocytes. After removing the supernatant, the resulting thymic fragments were subjected to two successive digestions with 125 µg/mL Liberase™ TL Research Grade low Thermolysin concentration (Sigma-Aldrich, Darmstadt, Germany) and 50 µg/mL DNase I grade II, from bovine pancreas (Roche Molecular Biochemicals, Basel, Switzerland) in DMEM at 37 °C for 15 min, with pipetting in the middle of the incubation period. The resulting supernatants were pooled and centrifuged. The resulting cell pellets were resuspended in 6 mL Percoll (1.07 g/mL), and DMEM was gently overlayed over Percoll and centrifuged at 500× *g* for 30 min at 4 °C with the brake off [30]. Interphase cells, which contain APC including TECs, were collected and, after adding 5 mL of DMEM with 2% FCS, centrifuged at 500× *g* for 5 min at 4 °C [31]. Cell pellets were resuspended in culture medium and submitted to TECs enrichment using the MagniSort™ Mouse CD45 Positive Selection Kit (Invitrogen, Waltham, MA, USA). Briefly, up to 10^8^ cells/100 µL were incubated with 20 μL of MagniSort^TM^ Anti-Mouse CD45 Biotin B for 10 min at room temperature. After that, 4 mL of cell separation buffer (PBS supplemented with 2% FCS, 1% penicillin, and streptomycin) was added and cells were washed by centrifugation at 500× *g* for 5 min. Cell pellets were resuspended and incubated for 10 min at room temperature with 20 μL of MagniSort^TM^ Positive Selection Beads A. Up to 2.5 mL of cell separation buffer were added, and the tube was inserted into the 6-Tube Magnetic Separation Rack (Cell Signal, Denvers, MA, USA) and incubated for 5 min at room temperature. The supernatant containing CD45^-^ cells was collected and washed twice by centrifugation at 500× *g* for 5 min.

For immunostaining, isolated TECs were resuspended in cell separation buffer and incubated with anti-CD16/CD32 Fc block 1:50 (clone 93, eBioscience, Bleiswijk, The Netherlands) during 15 min at 4 °C. Cells were then incubated with anti-CD45 FITC 1:100 (clone 104, BD biosciences, Allschwil, Switzerland) and anti-EpCAM/CD326 APC 1:100 (clone G8.8, Biolegend, San Diego, CA, USA) during 30 min at 4 °C. Resulting preparations contained about 80% of CD45^−^EpCAM^+^ cells (the reason we preferred to use the term enriched TECs instead of isolated or purified) as determined by flow cytometry analysis (data not shown).

### 2.5. RNA Extraction

Total RNA was extracted from washed thymuses and from enriched TECs by acid guanidium thiocyanate-phenol-chloroform extraction procedure using Tri-Reagent (Sigma, St. Louis, MO, USA), precipitated with isopropanol, and washed with 75% ethanol, as described by Chomczynski and Sacchi [32]. Sterile nuclease-free water subjected to the same extraction procedure served as negative control. For each kind of gene amplification, appropriate positive controls (cited below) were also enrolled in each experiment. RNA was submitted to DNase digestion, at 37 °C for 15 min, by DNase type I (Roche, Basel, Switzerland) to eliminate contaminating genomic DNA, followed by enzyme heat inactivation, at 85 °C for 8 min [33]. Purified RNA was dissolved in 30 µL of nuclease-free water (Ambion, Bleiswijk, The Netherlands), quantified using the Nanodrop 2000 (UV-Vis Spectrophotometer; ThermoScientific, Waltham, MA, USA) and stored at −80 °C until use in reverse transcription (RT).

### 2.6. Complementary DNA (cDNA) Synthesis

Total RNA was directly converted to cDNA using the M-MLV Reverse Transcriptase (RT, Invitrogen, Waltham, MA, USA). cDNA synthesis was performed with approximately 500 ng of RNA in a final volume of 20 µL containing 20 U/µL of RT, 2.5 µM of anchored-oligo(dT)_18_ primer (Roche, Basel, Switzerland), 60 µM of random hexamer primer (Roche), 20 U of RNase inhibitor (Promega, Walldorf, Germany), and 1 mM each deoxynucleoside triphosphate (dNTPs, Promega, Walldorf, Germany).

According to the manufacturer’s protocol, secondary structures were denatured by heating samples for 10 min at 65 °C in the presence of anchored-oligo(dT)_18_ primer, random hexamer primer, dNTP mix, and water PCR grade in a final volume of 20 µL. Samples were then cooled on ice immediately, then supplemented with 5X First-Strand Buffer, 0.1 M dithiotrethol (DTT) and 40U of RNaseOUT™ Recombinant Ribonuclease Inhibitor, and incubated at 37 °C for 5 min. One microliter (200 U) of M-MLV RT was added and each sample was incubated at 25 °C for 10 min followed by 50 min at 37 °C. Finally, the enzyme was inactivated by heating at 70 °C for 15 min. Two negative controls were performed in each reaction: one without RNA and the other without enzyme (RT minus control allowing the detection of eventual genomic DNA contamination).

### 2.7. Viral Load Quantification

To measure the viral load in whole thymuses and enriched TECs, 1 µL of cDNA was used to quantify CV-B4 RNA by qPCR, which was performed with 2× Takyon™ No Rox SYBR^®^MasterMixdTTP Blue (Eurogentec, Seraing, Belgium). The primer sequences used were: forward primer EV1 5′-CAAGCACTTCTGTTTCCCCGG-3′ and reverse primer EV2 5′-ATTGTCACCATAAGCAGCCA-3′ [34]. AMPLIRUN^®^ENTEROVIRUS 71 RNA CONTROL (Vircell, Granada, Spain) was used as a standard for a calibration curve containing 5-point ranging from 1.26 × 10^4^ to 1.26 copies/mL.

Each reaction consisted of 20 μL containing 1 μL of cDNA, 10 µL of TakyonMasterMix, and 3 pmol of each primer. qPCR was run on iCycleriQ real-time detection system (Bio-Rad, Hercules, CA, USA) using SYBR green detection with the following parameter: initial denaturation at 95 °C for 10 min, followed by 40 cycles of (denaturation at 95 °C for 30 s, annealing at 60 °C for 30 s, and elongation at 72 °C for 25 s). A melting curve from 55 to 95 °C was performed in each PCR reaction. Cyclethreshold (Ct) was defined as the PCR cycle number that crosses an arbitrarily placed signal threshold.

### 2.8. Viral Genome Detection in Enriched TECs

The PCR was carried out with 3 µL of cDNA from TECs samples and 0.4 µM of each primer 007 5′-ATTGTCACCATAAGCAGCCA-3′ and 008 5′-GAGTATCAATAAGCTGCTTG-3′, generating a 414 base pairs (bp) fragment [19], in a total volume of 50 µL containing 1.25 U of GoTaq G2 Flexi DNA polymerase (Promega, Walldorf, Germany), 0.2 mM each dNTP, and 2 mM MgCl_2_. The PCR mixture was subjected to a first denaturation step for 3 min at 94 °C, followed by 30 cycles of amplification, consisting of denaturation for 20 s at 94 °C, annealing for 20 s at 60 °C, and extension for 30 s at 72 °C, followed by a final extension step for 5 min at 72 °C.

PCR products were subjected to a subsequent semi-nested PCR with internal sense primer 006 5′-TCCTCCGGCCCCTGAATGCG-3′ and anti-sense 007 generating a 155 bp fragment [35]. A positive control (DNA amplified from the RNA extract of supernatant of CV-B4 E2-infected HEp-2 cells) and a negative control (no DNA) were included in each reaction.

### 2.9. Car Expression

For *Car* transcripts detection in enriched TECs, 1 µL of cDNA was incorporated to the same mixture and submitted to the same program as for viral genome detection by using *Car* primers (Table 1). cDNA from the heart was used as a positive control.

### 2.10. qPCR Assay

qPCR for transcription factors and autoantigens expression was performed using 2X Takyon™ No Rox SYBR^®^ MasterMixdTTP Blue (Eurogentec, Seraing, Belgium) with the same program as described for CV-B4 RNA quantification. Each reaction consisted of 20 μL containing 1 μL of cDNA, 10 µL of TakyonMasterMix, and 3 pmol of each specific pair of primers, *Aire*, *Fezf2*, *Igf2,* and *Myo7*, from Eurogentec (Table 1).

Each PCR reaction also included a reverse transcription negative control (without RT) to confirm the absence of genomic DNA and a no-template negative control to check for primer-dimers.

mRNA levels were normalized to those of *Oaz1*, recently identified as the most stable housekeeping gene in our experimental model [30].

Relative gene expression was calculated as follows [36]:

Relative gene expression = 2^−ΔΔCt^

With ΔΔCt = ΔCt_infected_ − ΔCt_control_

And ΔCt = Ct_target gene_ − Ct*_Oaz1_*

### 2.11. Western Blotting Analysis

Total proteins were extracted from the thymus with 200 µL RIPA buffer (Thermo Scientific, Waltham, MA, USA) supplemented with completed proteinase inhibitor cocktail (Pierce, Waltham, MA, USA). Lysates were incubated at 4 °C for up to 1 h with swirling, then centrifuged to remove DNA and cell debris at 12,000× *g* during 30 min at 4 °C. Protein concentrations were measured by bicinchoninic acid (BCA) protein assay kit (Pierce, Waltham, MA, USA).

Twenty micrograms of total proteins were loaded on 5–12% SDS gel electrophoresis and transferred to nitrocellulose membrane (Amersham Hybond, Darmstadt, Germany). The membranes were blocked for 1 h with 5% skim milk powder (Sigma, St. Louis, MO, USA) diluted in Tris-buffered saline with 0.1% Tween-20 (TBS-T).

Membranes were cut horizontally to detect each protein separately. The blots were incubated overnight with primary antibodies against VINCULIN, AIRE, FEZF2, MYO7, and IGF2 at 4 °C, with gentle agitation (Table 2). Blots were then incubated for 1 hour with secondary antibody, Anti-rabbit IgG HRP-linked antibody (Cell Signaling Technology, Leiden, The Netherlands).

The bands corresponding to the tagged proteins were detected using chemiluminescence (SuperSignal West Femto Maximum Sensitivity Substrate, Thermo Scientific, Waltham, MA, USA) and acquired on ImageQuant289 LAS4000 (GE Healthcare, Machelen, Belgium). Quantification of band intensity was performed with the ImageJ software. VINCULIN was used as loading control and for relative quantification.

### 2.12. Statistical Analysis

Statistical analysis was performed with GraphPad Prism 5 software (San Diego, CA, USA). The unpaired *t*-test was used for expression level (fold change) analysis in CV-B4- vs. mock-infected samples. *p* value < 0.05 was considered to represent a statistically significant difference. * *p* value < 0.05, ** *p* value < 0.01, *** *p* value < 0.001, and **** *p* value < 0.0001. The Spearman’s correlation test was used to analyze the correlation between the viral load and autoantigens expression in whole thymus in a given time point. Spearman’s *r* > 0: positive correlation; Spearman’s *r* < 0: negative correlation. To correct for multiple testing, data were then analyzed using the Benjamini-Hochberg (BH) test, which is defined by *P*_BH_ = p*nbp/j, where *p* is the original (uncorrected) *p*-value, nbp is the number of computed *p*-values in total, and j is the rank of the original *p*-value (when *p*-values are sorted in ascending order).

## 3. Results

### 3.1. In Utero CV-B4 Infection of Offspring’s Thymuses

#### 3.1.1. Viral Load in Whole Thymus

**Figure 2:**
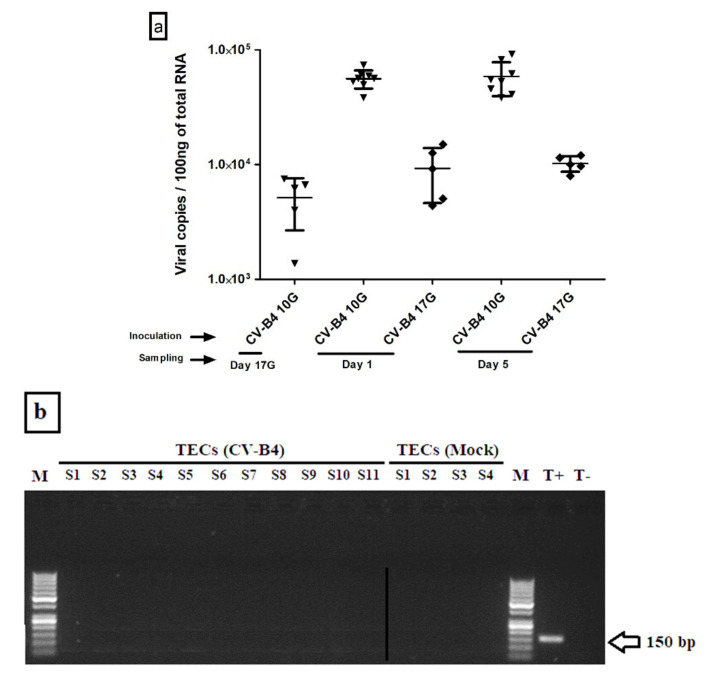

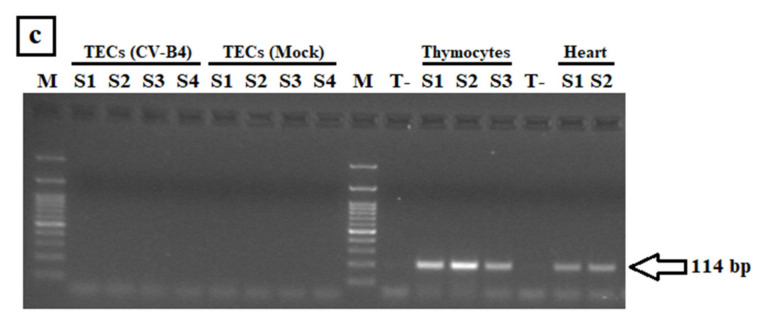
CV-B4 RNA quantification and detection in whole thymus and enriched TECs. (**a**). Viral RNA load in the whole thymus of offspring from CV-B4 E2-inoculated dams. Thymuses collected at different time-points (*n* = 6–13) were submitted to RNA extraction and then to viral RNA quantification by *RT-qPCR*. Results are expressed as copy numbers/100 ng of total RNA and represented as means ± SD. CV-B4 10G (▼): thymus harvested from mice born to dams inoculated with CV-B4 at Day 10 of gestation; CV-B4 17G (♦): thymus harvested from mice born to dams inoculated with CV-B4 at Day 17 of gestation. (**b**). Gel electrophoresis for CV-B4 RNA detection in enriched TECs by semi-nested PCR. (**c**). Gel electrophoresis for *Car* transcripts detection in enriched TECs, thymocytes and heart by PCR. M: Molecular weight ladder (100 bp); TECs (CV-B4): Enriched TECs harvested from in utero infected offspring; TECs (Mock): Enriched TECs harvested from age-matched controls; S: Sample; T+: Positive control (CV-B4 RNA); T−: Negative control (water PCR grade).

Before addressing the issue of genes expression, we first began by checking the infection in each sampled offspring’s thymus. The intensity of the infection was evaluated through measurement of CV-B4 RNA copy numbers in the different harvested offspring’s thymuses. Our results showed that when the virus inoculation was performed at day 10G, 82% of thymuses were positive, while about only 46% of thymuses were positive following inoculation at day 17G. Here, we should underline that only those samples positive for viral RNA were included in the analysis of genes expression compared to mock-infected controls. Then, the intensity extent of the infection was evaluated through measurement of CV-B4 RNA copy numbers in the different harvested offspring’s thymuses. Most elevated viral loads, ranging between 4 × 10^4^ and 9 × 10^4^ copies per 100 ng of total RNA, were recorded in samples at day 1 and 5 from offspring born to dams inoculated at day 10G (Figure 2a). Thymuses harvested from fetuses presented a lower level of CV-B4 RNA, ranging from 2073 to 8215 copies, comparable to those from offspring born to dams inoculated at day 17G and harvested at day 1 (ranging from 4373 to 15,040) or 5 (ranging from 8000 to 12,000).

#### 3.1.2. CV-B4 RNA Detection in Enriched TECs

Viral load in enriched TECs was equally quantified by RT-qPCR, but no trace of virus infection could be detected. Then, we used semi-nested PCR for its higher sensitivity but, as shown in Figure 2b, we still failed to detect CV-B4 RNA.

The expression of *Car*, the common receptor for CV-B and adenovirus, was assessed in enriched TECs (as well as in whole thymus and thymocytes) in an attempt to explain their supposed non-infectability. Indeed, *Car* transcripts were detected in whole thymus, thymocytes, and heart (used as a positive control), but not in enriched TECs (Figure 2c).

### 3.2. Transcription Factors and Autoantigens Transcripts in Whole Thymus

A couple of transcription factors (*Aire* and *Fezf2)* and autoantigens (*Igf2* and *Myo7*) were then selected for RNA quantification by RT-qPCR in mock-infected and confirmed CV-B4-infected offspring’s thymuses.

**Figure 3:**
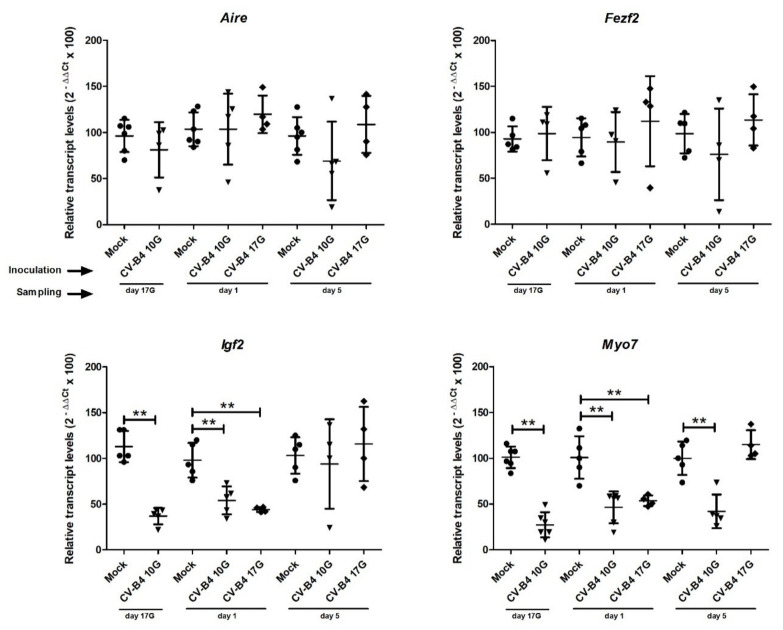
Transcription factors and autoantigens transcripts in the whole thymus. Complementary DNA obtained from mock- and CV-B4-infected thymuses (*n* = 5–7), harvested at different time-points, was submitted to quantification of *Aire*, *Fezf2*, *Igf2,* and *Myo7* transcripts by qPCR. Transcripts levels were normalized to those of *Oaz1.* Relative gene expression in CV-B4 E2- vs. mock-infected thymuses was calculated, as described in Section 2, and expressed as 100 × mean 2^−ΔΔCt^ values ± SD. The unpaired *t*-test was used for statistical analysis. ** *p* < 0.01. Mock (●) mock-infected thymuses harvested at different time points, from fetuses at day 17 of gestation (day 17G), and newborns at day 1 and day 5 from birth. CV-B4 10G (▼): thymus harvested from mice born to dams inoculated with CV-B4 at day 10 of gestation; CV-B4 17G (♦): thymus harvested from mice born to dams inoculated with CV-B4 at day 17 of gestation.

As illustrated in Figure 3, in utero CV-B4 infection did not induce any significant change in the level of *Aire* and *Fezf2* transcripts. Virus inoculation at day 10G led to decreased amount of *Aire* transcripts at day 17G and day 5, and of *Fezf2* at day 5, but variations were not significant.

On the contrary, a significant decrease in the level of *Igf2* transcripts was observed at day 17G (*p* = 0.0058) and day 1 (*p* = 0.004), following CV-B4 inoculation at day 10G, and at day 1 (*p* = 0.0079) following CV-B4 inoculation at day 17G.

Similarly, for *Myo7*, a significant decrease in transcripts level was detected at day 17G (*p* = 0.0025), day 1 (*p* = 0.0022), and day 5 (*p* = 0.008), following CV-B4 inoculation at day 10G, and only at day 1 (*p* = 0.0079) following virus inoculation at day 17G.

### 3.3. Transcription Factors and Autoantigens Transcripts in Enriched TECs

**Figure 4:**
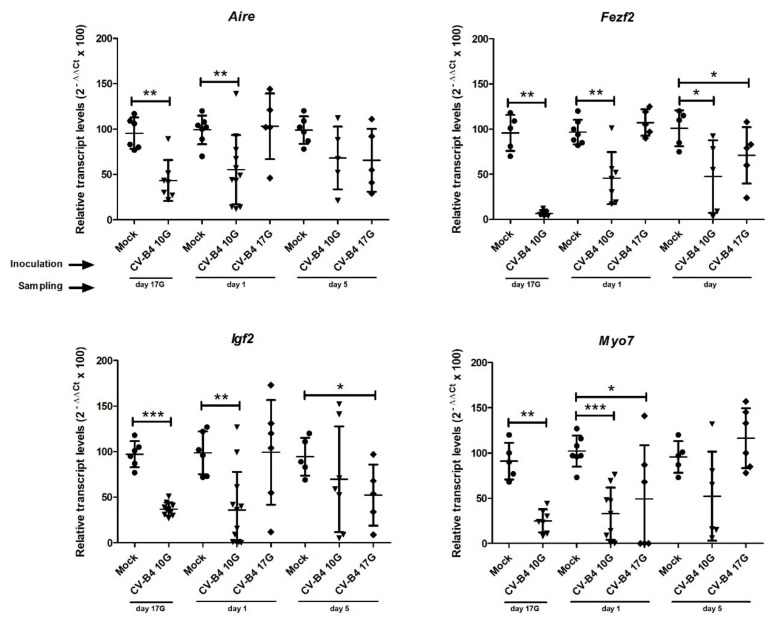
Transcription factors and autoantigens transcripts in enriched thymic epithelial cells. Whole thymuses were harvested, at different time-points, from offspring born to mock- and CV-B4-infected dams. Thymuses from fetuses or neonates of the same litter (*n* = 5–12) were pooled and subjected to TECs isolation. Transcripts levels for *Aire*, *Fezf2*, *Igf2,* and *Myo7*, in enriched TECs, were determined by RT-qPCR and normalized to those of *Oaz1.* Relative gene expression in TECs from CV-B4 E2- vs. mock-infected thymuses was calculated, as described in Section 2, and expressed as 100 × mean 2^−ΔΔCt^ values ± SD. The unpaired *t*-test was used for statistical analysis. * *p* < 0.05, ** *p* < 0.01, *** *p* < 0.001. Mock (●) TECs from mock-infected thymuses harvested at different time points, from fetuses at day 17 of gestation (day 17G), and newborns at day 1 and day 5 from birth. CV-B4 10G (▼): TECs from thymus harvested from mice born to dams inoculated with CV-B4 at day 10 of gestation; CV-B4 17G (♦): thymus harvested from mice born to dams inoculated with CV-B4 at day 17 of gestation.

Then, we chose to quantify transcripts of the same transcription factors and autoantigens in the cells in which they are expressed, namely TECs, which were obtained by purification and enrichment from whole thymuses (Figure 4).

Interestingly, our results showed a significant decrease in the level of *Aire* transcripts at day 17G (*p* = 0.0041) and day 1 (*p* = 0.0056) from birth, following inoculation at day 10G. A decrease in *Aire* transcripts was also observed at day 5 following inoculation either at day 10G or 17G, but differences were not significant compared to controls.

Similarly, *Fezf2* transcripts were significantly decreased at day 17G (*p* = 0.0013), day 1 (*p* = 0.0055), and day 5 (*p* = 0.0278), following virus inoculation at day 10G, but only at day 5 (*p =* 0.0476) following virus inoculation at day 17G.

For *Igf2*, a significant decrease was noted at day 17G (*p* = 0.0001) and day 1 (*p* = 0.0057), following inoculation at day 10G. Following inoculation at day 17G, that decrease became significant only at day 5 (*p* = 0.0278) from birth. 

As concerns *Myo7* transcripts, they were significantly decreased at day 17G (*p* = 0.0013) and day 1 (*p* = 0.0007), following virus inoculation at day 10G, and at day 1 from birth (*p* = 0.0369) when the virus was inoculated at day 17G.

### 3.4. Western Blot Analysis for Transcription Factors and Autoantigens Proteins

**Figure 5:**
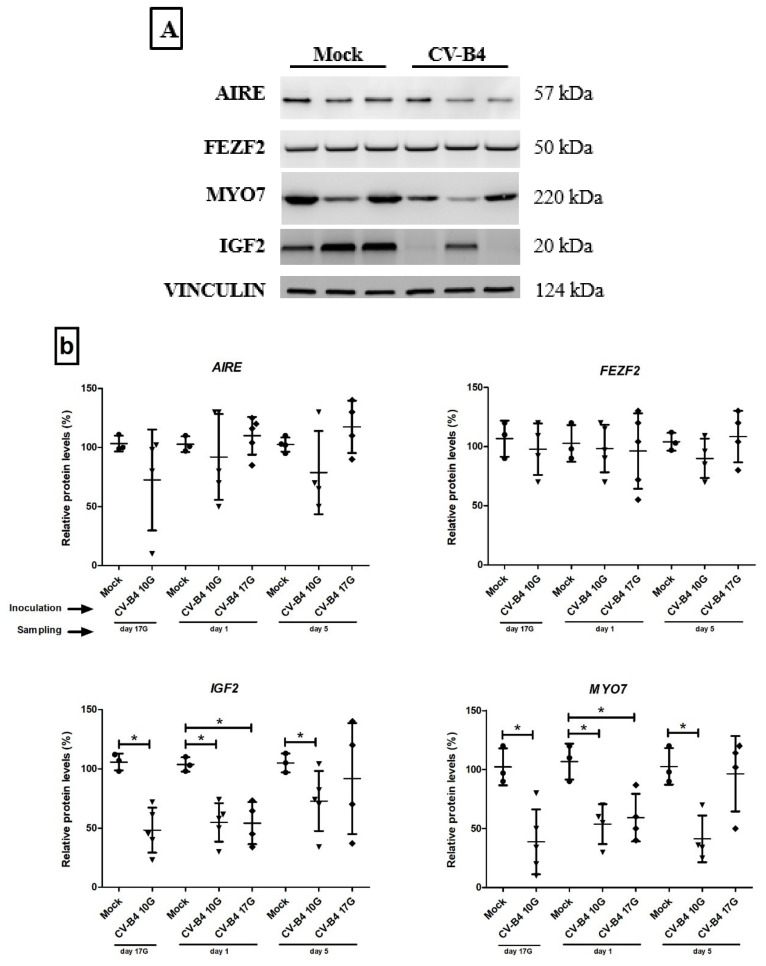
Transcription factors and autoantigens protein expression in the whole thymus. (**a**) Representative blots depicting the expression levels of AIRE, FEZF2, IGF2, and MYO7 proteins in CV-B4- and mock-infected thymus sampled at day 1. (**b**) Relative expression of AIRE, FEZF2, IGF2, and MYO7 determined by Western blot analysis. Proteins obtained from mock- and CV-B4-infected thymuses (*n* = 3–5), harvested at different time-points, were submitted to AIRE, FEZF2, IGF2, and MYO7 quantification by Western-blot analysis. Proteins levels were normalized to those of VINCULIN used as a loading control. Relative protein levels in CV-B4 E2- vs. mock-infected thymuses are represented as mean ± SD. The unpaired *t*-test was used for statistical analysis. * *p* < 0.05. Mock *(●):* mock-infected thymuses harvested at different time points, from fetuses at day 17 of gestation (day 17G), and newborns at day 1 and day 5 from birth. CV-B4 10G (▼): thymus harvested from mice born to dams inoculated with CV-B4 at day 10 of gestation; CV-B4 17G (♦): thymus harvested from mice born to dams inoculated with CV-B4 at day 17 of gestation.

Relative protein levels of AIRE, FEZF2, IGF2, and MYO7 were investigated by Western Blot in whole thymus as shown in Figure 5.

Analysis revealed that relative AIRE and FEZF2 protein levels did not change following infection compared to mock-infected thymuses. 

Relative IGF2 protein levels were, however, significantly lower at day 17G (*p =* 0.0179), day 1 (*p* = 0.018), and day 5 (*p* = 0.0357), following inoculation at day 10G, and at day 1 (*p* = 0.0286) following inoculation at day 17G compared to those in age-matched negative controls. Relative IGF2 protein level seems also decreased in thymuses sampled at day 5, following inoculation at day 17G, but the difference was not significant.

Similarly, the relative MYO7 protein levels were significantly decreased following inoculation at day 10G at different times of sampling (day 17G, *p* = 0.0179; day 1, *p* = 0.0286; and day 5, *p* = 0.029), and only at day 1 (*p* = 0.0286) following inoculation at day 17G.

### 3.5. Correlation between Viral Load and Transcription Factors and Autoantigens Transcripts Levels in the Thymus

To better assess the effect of CV-B4 infection on the expression of our selected autoantigens and transcription factors, we explored an eventual correlation between the observed variations and the viral load, by using Spearman’s correlation test. Multiple comparisons were corrected by means of the Benjamini–Hochberg method.

**Figure 6:**
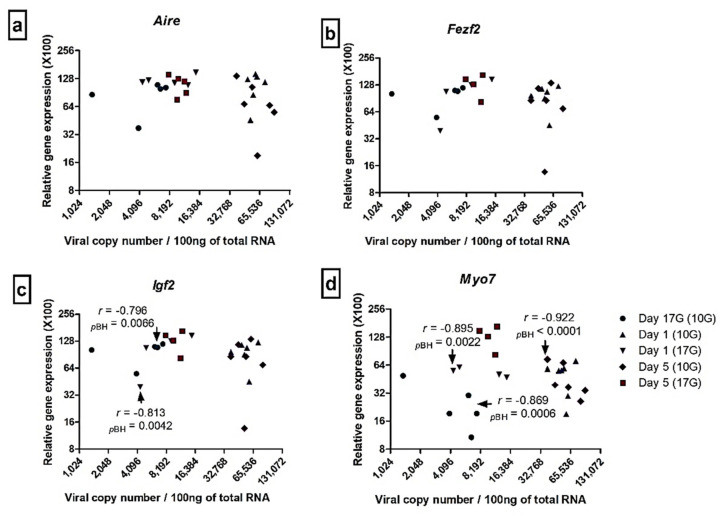
Relationship between viral load and transcription factors, *Aire* (**a**) and *Fezf2* (**b**), and autoantigens transcripts, *Igf2* (**c**) and *Myo7* (**d**), in the thymus. For each harvested thymus (CV-B4-infected or age-matched control), transcription factors and autoantigens transcripts together with the corresponding viral load were measured and plotted in the same graph. To better assess the effect of infection on transcription factors and autoantigens expression, an eventual correlation between both parameters was evaluated using the Spearmen’s correlation test. *r* is the Spearman’s correlation coefficient. *p*-values were corrected according to the Benjamini–Hochberg procedure (*P_BH_*). Thymuses harvested at day 17G (Day 17G (10G) (●)), day 1 (Day 1 (10G) (▲)) or day 5 (Day 5 (10G) (♦)) from mice born to dams inoculated (or mock) with CV-B4 at day 10 of gestation; thymuses harvested at day 1 (Day 1 (17G) (▼)) or day 5 (Day 5 (17G) (■)) from mice born to dams inoculated (or mock) with CV-B4 at day 17 of gestation. x and y axis were log-scaled.

As illustrated in Figure 6, no correlation was shown between viral load and *Aire* and *Fezf2* level expression. A negative correlation (Spearman’s *r* < 0) was observed between viral load and *Igf2* transcripts level at day 17G (*r* = −0.796, *P_BH_* = 0.0066) following inoculation at day 10G, and at day 1 (*r* = −0.813, *P_BH_* = 0.0042) following inoculation at day 17G. Similarly, a negative correlation was observed between viral load and *Myo7* transcripts level at day 17G (*r* = −0.869, *P_BH_* = 0.0006) and day 5 (*r* = −0.922, *P_BH_* < 0.0001), following inoculation at day 10G, and at day 1 (*r* = −0.895, *P_BH_* = 0.0022) following inoculation at day 17G.

## 4. Discussion

The incidence of autoimmune diseases is continuous and rapidly increasing worldwide, hence the need to multiply the efforts to fight this major health problem. The implementation of preventive and therapeutic strategies requires a good comprehension of the different factors and pathophysiological mechanisms behind each pathology. Our team has a long-standing (nearly twenty years) interest on the role of enteroviruses, especially CV-B, shown as major environmental triggers, in the pathogenesis of T1D. We were essentially working on the elucidation of the pathophysiological mechanisms of CV-B4 infection leading to T1D. Infection of the thymus, as the central site of immune self-tolerance establishment, is one of the most important hypotheses we are exploring. As mentioned in the introduction, results obtained from our investigations performed in vitro strongly confirmed our hypothesis [21,22,23,24,25]. We also showed that CV-B4 can infect and even persist in the thymus during the course of a systemic infection of outbred mice inoculated by the oral route [19]. The ability of CV-B4 to infect the thymus in utero, a period during which the thymus most actively fulfils its functions of T cell education and maturation, was, however, the remaining question.

A few years ago, we described how CV-B4 E2 inoculation of *Swiss albino* mice at day 10G or day 17G has an effect on pregnancy outcome and on offspring [37]. In addition, CV-B4 can reach offspring’s thymus and disturb T cell differentiation [26]. Hence, we felt this is the ideal model to pursue our investigations about the role of CV-B4 infection of the fetal and neonatal thymus in the genesis of autoimmunity. The current paper addresses the issue of an eventual effect of in utero CV-B4 infection on the expression of selected autoantigens and transcription factors in the thymus.

Autoantigens, also referred to as promiscuous genes, tissue-specific self-antigens or tissue-restricted antigens (TRA), are essential in the process of establishment and maintenance of central tolerance. TRA are promiscuously expressed by medullary TECs [36] and presented to thymocytes to be educated. In fact, during the central tolerance establishment process, in fetal and neonatal life, immature T cells are educated to recognize and discriminate foreign antigens from the body’s own components. T cells recognizing autoantigens are eliminated through a mechanism known as negative selection of autoreactive cells. *Aire* or and *Fezf2* are two transcriptional controllers that drive the expression of many TRA [37].

It is obvious that we had to begin our investigation by checking thymus infection before addressing the issue of genes expression. For a more accurate analysis, we chose to include only samples positive for viral RNA. As previously reported [26], vertically transmitted CV-B4 reached an important proportion of offspring’s thymuses, especially following inoculation at day 10G. In addition, viral load assessment by RT-qPCR showed higher values following inoculation at day 10G than at day 17G.

Conversely, enriched TECs showed negative results for viral RNA detection, either by RT-qPCR or RT-semi-nested-PCR. We thought that TECs infection would have occurred and left quickly after inoculation, before the first sampling that was performed 7 days after inoculation at day 10G and about 4 days after inoculation at day 17G. Nevertheless, we failed to detect CV-B4 RNA in the thymus of fetuses harvested 48 h after inoculation (data not shown). In a recent study, viral RNA was detected only in the pancreas of CV-B4-inoculated Swiss mice, but not in sorted total thymic cells isolated after enzymatic digestion [38].

Hence, we wondered if those TECs express the specific receptor for CV-B4, namely CAR (Coxsackievirus and Adenovirus Receptor), the common receptor for CV-B1 to 6 and adenovirus 2 and 5 [39]. *Car* transcripts were detected in heart (used as a positive control), whole thymus, thymocytes, and in cells of the interphase following Percoll isolation, but not in TECs obtained after enrichment by CD45 Positive Selection kit. We speculate that cells other than TECs were *Car^+^* in the interphase since the latest contains heterogeneous cells known as APC [31]. The absence of *Car* expression in those TECs is in favor of their non-infectability and, therefore, may explain the failure to detect CV-B4 RNA. CV-B4 infection of neonatal murine TECs was, however, previously documented, but it was observed in vitro, in a continuous cell line (MTE4–14) of medullar origin derived from an inbred mouse strain (C3H/J) [23]. Nevertheless, we cannot totally exclude the presence of CAR and viral RNA in our TECs since any detection method, regardless of its sensitivity, has limits. Moreover, the behaviour of viruses in primary cells is poorly understood. Viruses can undergo changes in primary cells (such as loss of sequences) that can further interfere with their detection.

Then, we evaluated the expression of selected autoantigens and transcription factor in both total thymus and enriched TECs. Autoantigens, also referred to as promiscuous genes, tissue-specific self-antigens or tissue-restricted antigens (TRA), are essential in the process of establishment and maintenance of central tolerance. TRA are promiscuously expressed by medullary TECs [40] and presented to thymocytes to be educated. In fact, during the central tolerance establishment process, in fetal and neonatal life, immature T cells are educated to recognize and discriminate foreign antigens from the body’s own components. T cells recognizing autoantigens are eliminated through a mechanism known as negative selection of autoreactive cells.

Gene expression was analyzed until day 5 after birth, since most autoantigens are expressed in the thymus at the highest level during the embryonic period and expression declines few days after birth [41]. As mentioned below, TRA expression during that period is essential for the establishment of long-lasting self-tolerance [42].

For qPCR analysis, we used *Oaz1* as an internal control gene, as we recently demonstrated that it is the most stable in the thymus, among other tested housekeeping genes, during development of *Swiss albino* mice and following their in utero infection by CV-B4 [30].

Until now, only two independent transcriptional factors, AIRE and FEZF2, regulated by distinct signaling pathways and promoting the expression of different classes of proteins, have been recognized to orchestrate the expression of a wealth of TRA in medullary TECs [7,43,44]. *Aire* is a member of the zinc-finger gene family, whose expression is regulated by Tumor Necrosis Factor (TNF) family members, RANK, and CD40 signaling pathway, and that indirectly/epigenetically regulates *Aire*-dependent TRA through AIRE interaction with nuclear proteins [44]. *Aire* mutation induces autoimmune polyglandular syndrome type 1 in patients and *Aire* knockout mice [45,46]. *Fezf2*, also known as *Zfp312* and *Fezl*, expression is however under regulation of Lymphotoxin β receptor (LTβR) signaling axis, and directly regulates *Fezf2*-dependent TRA through FEZF2 binding on the gene promoter. Mice lacking *Fezf2* in mTECs displayed severe autoimmune symptoms [42]. In humans, no association has been reported between *Fezf2* mutations and autoimmune disease, but rather with symptoms like autism and neoplastic diseases [47,48,49].

Regarding the choice of the autoantigens to be analyzed, we took into account that CV-B4 E2 is commonly known as a diabetogenic strain, but it has also been shown to target several tissues [19,20]. However, since the most frequent autoimmune manifestations involving CV-B concern pancreas and heart (reviewed in [26]), one autoantigen representative of each of those two tissues was retained, namely *Igf2* and *Myo7*, respectively.

*Igf2* belongs to the insulin family of polypeptide growth factors and constitutes the dominant polypeptide, inside that family, to be found in the thymus [50]. A genetic defect in intrathymic expression of *Igf2* is associated with autoimmune diabetes in BBDP (*BioBreeding Diabetes Prone*) rats, which may contribute to the absence of central T cell self-tolerance to the insulin hormone family [51]. Insulin-related peptides are under AIRE control and are transcribed in the murine mTECs according to a precise hierarchy (*Igf2* > *Igf1* > *Ins2* > *Ins1*). Such hierarchical profile suggests an association with a higher immunological tolerance to *Igf2* and a lower tolerance to insulin (reviewed in [52]).

Similarly, cardiac myosin is considered as the dominant autoantigen in autoimmune heart disease, mainly myocarditis and dilated cardiomyopathy. *Myo7* gene encodes the beta heavy chain subunit of cardiac myosin. Our choice of *Myo7* is based on its predominant expression in medullary TECs and other thymic cell subtypes [53].

At the practical level, gene expression was analyzed until day 5 after birth, since most autoantigens are expressed in the thymus at the highest level during the embryonic period and expression declines a few days after birth [42]. As mentioned below, TRA expression during that period is essential for the establishment of long-lasting self-tolerance [43].

For qPCR analysis, we used *Oaz1* as an internal control gene, as we recently demonstrated that it is the most stable in the thymus, among other tested housekeeping genes, during the development of *Swiss albino* mice and following their in utero infection by CV-B4 [30].

Results of quantification in the whole thymus showed a notable effect of in utero CV-B4 infection on the level of *Myo7* and *Igf2* transcripts, but not on that of *Aire* and *Fezf2*. *Ins2* and *Myo6* transcripts were also quantified, but not detected (data not shown). In fact, as mentioned above, *Ins2* is not a dominant member of the insulin family to be expressed in the thymus, and its expression in murine thymus begins after birth [42]. As regards *Myo6*, our results are in agreement with those of Lv et al. [53], who reported the absence of *Myo6* expression in the whole thymus and purified thymic cell subsets from B6, NOD, and DQ8^+^NOD mice. Similarly, an absence of *Myo6* expression in the human thymus was noted, vs. a high level in the heart [53,54].

Due to the expression of those genes being restricted to mTECs that represent a minority among total thymic cells [55], we judged that it would be more accurate to analyze their expression in enriched TECs. It was necessary to pool different thymuses from the same litter to obtain a sufficient number of cells for transcripts analysis. Two coupled techniques (Percoll and CD45 positive selection) were used to obtain the most pure TECs suspension. After TECs enrichment, the difference in expression compared to control mice became more evident. Indeed, relative amounts of *Aire* and *Fezf2* transcripts were found to be significantly diminished, especially following inoculation at day 10G. As regards *Igf2* and *Myo7* transcripts, they were also decreased following CV-B4 infection, with, in some points, more significant differences than those observed in the whole thymus.

Then, considering the fact that the infection may have an impact on different levels of host gene expression, mainly during mRNA synthesis [56,57] and/or during proteins production and maturation [58], the amount of proteins encoded by our four genes was assessed using Western blot. In line with RT-qPCR results, analysis at the protein level revealed a decrease in IGF2 and MYO7 amounts in the whole thymus, following CV-B4 E2 infection, with no significant effect on AIRE and FEZF2. Unfortunately, protein extraction from enriched TECs, or from the organic phase of Trizol extraction, generates a very low yield, which prevents us from performing Western blot on those samples. A decrease in relative amounts of *Igf2* transcripts and IGF-2 protein in CV-B4 E2-infected MTE4–14, a murine TEC line of medullar origin [59], was however already documented by our team [23].

Correlation analysis revealed that most of the observed variations in relative gene expression, especially following inoculation at day 10G, correlate negatively with the matched amount of RNA copies found in the whole thymus harvested at different time points. In other words, *Igf2* and *Myo7* transcripts level decreased with the increase of the viral load in the infected thymus.

The effect of CV-B4 in utero infection of the thymus on transcription factors and autoantigens expression, especially in TECs for which we could not prove the infection, might be indirect via epigenetic regulation [60,61,62]. Similar to our results, a downregulation of IGF2 expression in enriched TECs of mice following CV-B4 infection was observed, despite the absence of a proof of infection of those cells, which raises this hypothesis [38]. Indeed, demethylation of DNA was one of the suggested mechanisms of epigenetic control of autoantigens expression in murine mTECs, such as *Igf2* [63]. We should also note that *Aire* expression is regulated by RANK/CD40 pathways, while *Fezf2* is regulated by the LTβR pathway [44]. A loss in LTβR was reported as a cause of a reduction of *Fezf2* expression in mTECs [64]. Since *Lta*, *Ltb*, and *Light* are known as LTβR ligands [64], interference of CV-B4 infection with any of those regulation pathways may contribute to a disturbance in *Fezf2* and FEZF2-dependent TRA expression. It has been suggested that infection can alter signaling pathways via indirect effects such as the host-mediated immune (INF, Interleukin, TNF) and hormonal response [65]. Indeed, that indirect regulation may occur by mediators of the immune response against the infection, such as type I interferon (IFN)-α [66]. It has been reported that in vitro IFN-α or IFN-β treatment down-regulates *Igf2* expression [67,68,69]. Irradiated CV-B1 interaction with Toll-like receptors induces a normal production of IFN-α, IFN-β, and IFN-γ, suggesting that CV-B does not require replication within cells to induce IFNs expression [66].

Another putative mechanism is that, following infection, increases or decreases in certain microRNAs (miRNAs) are also known to interfere with gene expression, enhancing inflammatory T cell development and consequently promoting autoimmunity [70]. Interestingly, enterovirus infection may play a role in miRNAs alteration [71]. It has been shown that mTEC maturation, and *Aire*, *Fezf2,* and autoantigens expression are regulated by miRNAs [60]. TNF family cytokines, LTΒR, RANK, and CD40, were found as some of the potential target genes of miRNAs [63]. CV-B infection may interfere with those genes or genes coding for their ligands, leading to a dysregulation of FEZF2, AIRE, and autoantigens expression [72].

Finally, thymic crosstalk, known as the interaction between thymocytes and TECs, is essential for maturation and differentiation of both cell types [63]. Thymic crosstalk is mediated by TNF family cytokines expressed in thymocytes and their receptors expressed in mTEC [63]. In our recent studies, we noted a significant effect of CV-B4 in utero infection of the thymus on T cell maturation/differentiation and thymic output, which may interfere with thymocytes–TEC interactions, therefore, inhibiting mTEC functions, and mainly transcription factors and autoantigens expression [26,73].

## 5. Conclusions

In summary, this study underscores the pronounced effect of CV-B4 E2 in utero infection of mouse thymus, on the expression of selected transcription factors and autoantigens involved in tolerance establishment. A decrease in *Igf2* and *Myo7* expression was evident in infected thymuses at both the transcriptional and protein level. In addition to the decrease in *Igf2* and *Myo7* transcripts, a decrease in *Aire* and *Fezf2* transcripts was evident in enriched TECs. To the best of our knowledge, this is the first study evaluating transcription factors and autoantigens expression in the whole thymus and TECs in the context of any in utero infections. An important lack of *Aire* and *Fezf2* transcription factors would likely induce the development of AID. With regard to the defect in *Igf2* and *Myo7* expression, it is likely supposed to break tolerance to the pancreas (β cells) and heart (myocardium), respectively. Complementary studies are being conducted along this line in our laboratory, in an attempt to make a link between those observations and an eventual appearance of such suggested autoimmune manifestations in the long term.

## Figures and Tables

**Table 1:** Primers sequences for qPCR.

Gene	Forward Primer	Reverse Primer	Gene ID	Amplicon Length (bp)
*Oaz1*	5’-GCCAATGAACGAGATCACTT-3′	5′-GCTGTTTAAGATGGTCAGGTGA-3′	18245	110
*Aire*	5′-GGTTCTGTTGGACTCTGCCCTG-3′	5′-TGTGCCACGACGGAGGTGAG-3′	11634	144
*Fezf2*	5′-GTGGCTCCCACCTTTGTACATTCA-3′	5′-TCACGGTGACAGGCTGGGATTAAA-3′	54713	121
*Igf2*	5′-GGGAGCTTGTGGACACGC-3′	5′-GCACTCTTCCACGATGCCA-3′	16002	107
*Myo7*	5′-TGCAAAGGCTCCAGGTCTGAGG-3′	5′-GCCAACACCAACCTGTCCAAGT-3′	140781	203
*Car*	5′-GGTTTGAGCATCACTACACCCG-3′	5′-TTCAATGTCCAGTGGTCCCTGG-3′	13052	114

**Table 2:** Antibodies used for Western blot.

Antibody	Host Species	Dilution	Clone	Reference	Supplier	Tagged Protein Size (kDa)
VINCULIN	Rabbit	1/500	42H89L44	AB-2532280	Thermofisher Scientific	124
AIRE	Rabbit	1/1000	Polyclonal	PA5-24554	Thermofisher Scientific	57
FEZF2	Rabbit	2/1000	Polyclonal	A05051	Thermofisher Scientific	50
IGF2	Rabbit	1/500	OAAB07463	OAAB07463	BosterBio	20
MYO7	Rabbit	4/1000	Polyclonal	PA1-936	Aviva Systems Technology	220

## Data Availability

Data are contained within the article.
